# Decision-making in percutaneous coronary intervention: a survey

**DOI:** 10.1186/1472-6947-8-28

**Published:** 2008-06-25

**Authors:** Catherine R Rahilly-Tierney, Ira S Nash

**Affiliations:** 1 Massachusetts Veterans Epidemiology and Research Information Center (MAVERIC), Boston VA Healthcare System, Boston, Massachusetts, USA; 2 Division of Aging, Brigham and Women's Hospital, Boston, Massachusetts, USA; 3 Zena and Michael A. Wiener Cardiovascular Institute, Mount Sinai Medical Center, New York, New York, USA

## Abstract

**Background:**

Few researchers have examined the perceptions of physicians referring cases for angiography regarding the degree to which collaboration occurs during percutaneous coronary intervention (PCI) decision-making. We sought to determine perceptions of physicians concerning their involvement in PCI decisions in cases they had referred to the cardiac catheterization laboratory at a major academic medical center.

**Methods:**

An anonymous survey was mailed to internal medicine faculty members at a major academic medical center. The survey elicited whether responders perceived that they were included in decision-making regarding PCI, and whether they considered such collaboration to be the best process of decision-making.

**Results:**

Of the 378 surveys mailed, 35% (133) were returned. Among responding non-cardiologists, 89% indicated that in most cases, PCI decisions were made solely by the interventionalist at the time of the angiogram. Among cardiologists, 92% indicated that they discussed the findings with the interventionalist prior to any PCI decisions. When asked what they considered the best process by which PCI decisions are made, 66% of non-cardiologists answered that they would prefer collaboration between either themselves or a non-interventional cardiologist and the interventionalist. Among cardiologists, 95% agreed that a collaborative approach is best.

**Conclusion:**

Both non-cardiologists and cardiologists felt that involving another decision-maker, either the referring physician or a non-interventional cardiologist, would be the best way to make PCI decisions. Among cardiologists, there was more concordance between what they believed was the best process for making decisions regarding PCI and what they perceived to be the actual process.

## Background

Interventional cardiologists are more likely to recommend percutaneous coronary intervention (PCI) than physicians of other specialties in cases of angiographically-determined coronary artery disease. [1-3] Differences in recommendations by specialty highlight uncertainty about the appropriateness of such interventions. [4] Given this uncertainty, some authors have called for formal or informal collaboration between physicians of different specialties, such as general internists, cardiologists, and cardiothoracic surgeons, in making PCI decisions. [5-7] Studies examining group processes in determining appropriateness of coronary procedures have found that physicians of diverse specialties are likely to converge on a given recommendation after discussion in a multi-specialty panel. Moreover, recommendations made through multi-disciplinary panel discussion are more consistent than those of individual physicians [8,9].

Few researchers have explored the perceptions of physicians referring cases for coronary angiography regarding the degree to which collaboration actually occurs during PCI decision-making. We sought to determine whether physicians referring their patients for coronary angiography perceived themselves as involved in PCI decisions. We also sought their opinion regarding whether collaboration between either themselves or a non-interventional cardiologist and the interventionalist is best during decision-making regarding PCI.

## Methods

We developed a brief survey (see Additional File 1) to elicit perceptions of internal medicine faculty who referred their patients to the cardiac catheterization laboratory for coronary angiography at the Mount Sinai Medical Center. Faculty were invited to participate with a mailing that included the survey and a cover letter explaining that their participation was voluntary and anonymous. We chose to use an anonymous survey so that responders would be more likely to answer questions honestly. To capture as many different kinds of referring physicians as possible, we attempted to contact all full and part-time internal medicine faculty, regardless of specialty. We gave potential participants approximately 2 months to return the survey. Mount Sinai's Internal Review Board deemed the anonymous survey exempt from their review.

Concordance between what responders perceived as the actual process by which PCI decisions are made, and what they believed was the ideal process by which such decisions are made, was established by comparing their responses to questions 4 and 5 on the survey, as follows. Responses were considered concordant if they indicated the interventionalist solely made PCI decisions *and *the responder felt this was the best process by which decisions are made, or if they indicated that they were involved in collaboration during revascularization decisions *and *felt collaboration was the best process for decision-making. Responses were considered discordant if they indicated they were not involved in collaboration *and *indicated they felt the best process would have been discussion between either themselves or a non-interventional cardiologist and the interventionalist prior to PCI.

Concordance of responses was established using only surveys in which the two questions required to establish concordance were answered. A t-test was used to determine whether the number of patients referred for angiography differed between concordant surveys and discordant surveys. Some responders selected more than one reponse for some questions; in most of these cases, responders chose two options that were similar to one another. For instance, some responders indicated they would prefer to involve a non-interventional cardiologist either to review the angiogram and discuss the case with the interventionalist, or only to discuss the case with the interventionalist. Such a response would be counted as preferring to involve a non-interventional cardiologist in the case.

## Results

Of 378 surveys mailed, 35% (133) were returned answered. Of 88 surveys mailed to general internists or medicine-pediatrics physicians, 42% (37) were returned. Of 166 surveys mailed to non-cardiology sub-specialists, 33% (55) were returned. Of 73 surveys mailed to cardiologists, 56% (41) were returned. Only one of these indicated that he or she is an interventional cardiologist; the remainder identified themselves as non-interventional cardiologists. The median year of graduation from medical school was 1984 and the range of year of graduation was 1948–2001. The median number of patients referred for coronary angiography was 6, with a wide range of zero to 500 patients.

As shown in Table 1, 58% (45 of 78) of responses from non-cardiologists were discordant. Among non-cardiologists who answered the question, 89% (71 of 80) indicated the interventionalist made PCI decisions without collaboration. However, of non-cardiologists who answered the question, 66% (58 of 88) indicated some collaboration between the interventionalist and another physician involved in the case would have been the best process. 48% (42 of 88) of non-cardiologists who answered the question indicated they would prefer a non-interventional cardiologist be involved to review the angiogram and/or discuss the case with the interventionalist, and 18% (16 of 88) indicated they would have preferred discussion between themselves and the interventionalist. Among non-cardiologists, there was no significant difference in number of patients referred for angiography between responses that were concordant and responses that were discordant (p-value 0.68).

**Table 1:** Concordance or discordance of responders, stratified by cardiology or non-cardiology specialty.

Specialty	Number of responses	Number concordant (%)	Number discordant (%)
Non-cardiology	78	33 (42%)	45 (58%)
Cardiology	39	36 (92%)	3 (8%)

Surveys with missing data for one or both of the questions used to determine concordance or discordance of a participant's responses are not represented here.

In contrast to non-cardiologists, 92% (36 of 39) of cardiologists who answered the question indicated they reviewed the angiogram and/or discussed the case with the interventionalist during PCI decision-making. 95% (37 of 39) of cardiologists who answered the question indicated that they felt such collaboration represents the best process by which PCI decisions are made. 92% of responses from cardiologists were thus concordant.

## Discussion

The results of this survey suggest that the majority of non-cardiologists and cardiologists in this academic medical center believe collaboration between two or more physicians involved in a case is the best way to make PCI decisions. Because cardiologists perceived themselves as included in PCI decision-making in cases they had referred for coronary angiography, their survey responses indicated concordance between what they believed was the best process for making decisions regarding PCI and what they perceived to be the actual process. Because non-cardiologists perceived that the interventionalist made PCI decisions without collaboration in many cases referred for coronary angiogram, a majority of their survey responses reflected discordance between what they percieved to be the actual and ideal process for making decisions regarding PCI.

There are several limitations to this survey study. First, only 35% of surveys mailed were returned completed, and responding physicians may not have been representative of all referring physicians. Because the survey was anonymous, we could not follow-up on surveys that were not returned. Our sample thus may reflect a disproportionate number of responses that were discordant. The survey was limited to internal medicine faculty within a single academic center. However, given that previous authors have called for collaboration between physicians of multiple specialties in the course of PCI decision-making, we expect these results are generalizable to other major academic medical centers. We did not ask responders directly whether or not they would be satisfied with PCI should a potentially intervenable lesion on angiography be found; such a question might have more directly elicited whether referring physicians agree or disagree with PCI decisions made during angiography. Finally, we designed the study to elicit perceptions of actual process of decision making, and opinions regarding the ideal process by which such decisions are made. Objectively determining the actual process by which revascularizations are made and which members of the medical team are directly involved in that process in the real world would be an important next step in exploring decision-making processes in PCI following angiography.

There are several potential reasons why referring cardiologists percieved themselves as more involved in PCI decision-making than referring non-cardiologists. As a single cardiologist cares for many patients with chronic ischemic heart disease, he or she is likely to have developed a professional relationship with catheterization laboratory staff. Cardiologists are more likely to be familiar with coronary anatomy and the significance of angiogram findings as a result of their specialty training. Therefore, the interventionalist on a case may be more likely to respect the opinion of a fellow cardiologist when discussing the results of angiography than the opinion of a non-cardiologist. The physical proximity of the interventional and non-interventional cardiologists at the time an angiogram may facilitiate conference regarding any intervention being considered.

In contrast to cardiologists, non-cardiologists percieved that they were less involved in decision-making. This may represent their perception of their involvement, not their actual involvement in PCI decisions. In many of their referred cases, a non-interventional cardiologist was likely involved who referred patients directly to the catheterization laboratory and interacted with laboratory staff before and during the proceure. Of the non-cardiologists who indicated that they believed some other decision-maker should be involved in the process, 72% (42 of 58) who answered the question indicated involving a non-interventional cardiologist would be better than having the interventionalist make the decision alone. This interaction may indeed already be occurring without the referring non-cardiologist's awareness.

While it may not be reasonable to expect the interventionalist to interrupt the angiogram to contact a referring non-cardiologist in order to discuss intervention decisions, the number of discordant responses from non-cardiologists suggests that these responders would support some improvement to the process of PCI decision-making as it existed at the time of administration of this survey in this academic center. For example, if no non-interventional cardiologist is involved in a case scheduled for angiogram, a brief pre-procedure telephone conversation between the interventionalist and the primary care provider, during which the primary physician might express whether they would prefer a non-interventional specialist be involved in any required decision-making, may be feasible.

We did not address whether or not collaboration on PCI decisions improves outcomes. The results of the recent COURAGE trial indicate that risk of adverse cardiac outcomes in patients who undergo PCI or medical management for stable coronary disease is equivalent. [10] Because uncertainty surrounding appropriateness of PCI in patients referred for coronary angiography will persist, the majority of referring physicians we surveyed indicated that collaboration between the interventionalist and either a non-interventional cardiologist or the referring physician themselves is the best way to make PCI decisions.

## Conclusion

The results of this survey study indicate that most physicians referring patients for coronary angiography agree that a collaborative approach to PCI decision-making is best. Further studies might include multiple institutions, obtain a larger sample of physicians to survey, or explore the impact of collaboration on referring physician satisfaction or on patient outcomes.

## Competing interests

The authors declare that they have no competing interests.

## Authors' contributions

CRR–T and ISN participated in the conception and design of the survey. CRR–T distributed and collected the survey, compiled the data, and performed statistical analyses. CRR–T and ISN both drafted and edited the manuscript.

## Pre-publication history

The pre-publication history for this paper can be accessed here:



## Supplementary Material

Additional file 1 Survey employed in the study. The file provided is the actual anonymous survey mailed to 378 full- and part-time faculty at the medical center. Click here for file
