# Supplementary material for: Decision-making in percutaneous coronary intervention: a survey
Source: BMC Med Inform Decis Mak. 2008 Jun 25;8:28. doi: 10.1186/1472-6947-8-28 (PMC2443120; doi:10.1186/1472-6947-8-28)
Supplement: Additional file 1 — Survey employed in the study. The file provided is the actual anonymous survey mailed to 378 full- and part-time faculty at the medical center. [file 1472-6947-8-28-S1.doc]

APPENDIX. Survey employed in the study.

1. Which best describes your training?
   1. Internal Medicine
   2. Internal Medicine followed by a sub-specialty, not cardiology
   3. Internal Medicine followed by cardiology, not interventional cardiology
   4. Internal Medicine followed by interventional cardiology
   5. Family Practice or Medicine-Pediatrics
   6. Other; if so, describe _____________________________
2. Which year did you graduate from medical school? _______
3. About how many patients of yours underwent coronary angiography in the last 12 months? ___________
4. For patients of yours who had coronary angiography, which best describes the process by which decisions regarding revascularization were made?
   1. You reviewed the angiogram and discussed the findings with the interventional cardiologist, prior to any revascularization decisions and/or procedures
   2. You discussed the findings of the angiogram with the interventional cardiologist, prior to any revascularization decisions and/or procedures
   3. The interventional cardiologist made and/or executed revascularizations decisions at the time of the angiogram
5. What do you consider the best process for decisions regarding coronary revascularization?
   1. A referring non-cardiologist discusses the findings of the angiogram with the interventional cardiologist prior to any revascularization decisions
   2. A non-interventional cardiologist reviews the angiogram and discusses the findings with the interventional cardiologist, prior to any revascularization decisions
   3. A non-interventional cardiologist discusses the findings with the interventional cardiologist, prior to any revascularization decisions
   4. The interventional cardiologist makes and/or executes revascularization decisions at the time of the angiogram.
6. Use the space below to make any additional comments you would like regarding your utilization of the services of an interventional cardiologist, that were not elicited by the questions above.
